# How to develop a meaningful radiomic signature for clinical use in oncologic patients

**DOI:** 10.1186/s40644-020-00311-4

**Published:** 2020-05-01

**Authors:** Nikolaos Papanikolaou, Celso Matos, Dow Mu Koh

**Affiliations:** 1 Computational Clinical Imaging Group, Champalimaud Foundation, Centre for the Unknown, Av. Brasília, Doca de Pedrouços, 1400-038 Lisbon, Portugal; 2 Department of Radiology, Champalimaud Centre for the Unknown, Lisbon, Portugal; 3 Department of Radiology, Royal Marsden Hospital, Sutton, UK

**Keywords:** Radiomics, Machine learning, Quantitative imaging

## Abstract

During the last decade, there is an increasing usage of quantitative methods in Radiology in an effort to reduce the diagnostic variability associated with a subjective manner of radiological interpretation. Combined approaches where visual assessment made by the radiologist is augmented by quantitative imaging biomarkers are gaining attention. Advances in machine learning resulted in the rise of radiomics that is a new methodology referring to the extraction of quantitative information from medical images. Radiomics are based on the development of computational models, referred to as “Radiomic Signatures”, trying to address either unmet clinical needs, mostly in the field of oncologic imaging, or to compare radiomics performance with that of radiologists. However, to explore this new technology, initial publications did not consider best practices in the field of machine learning resulting in publications with questionable clinical value. In this paper, our effort was concentrated on how to avoid methodological mistakes and consider critical issues in the workflow of the development of clinically meaningful radiomic signatures.

## Background

In the past decades, medical imaging has proven to be a useful clinical tool for the detection, staging, and treatment response assessment of cancer. Consequently, conventional imaging is often viewed as “old” technology, a misperception that unfortunately, has perhaps limited the harnessing of the full potential of medical images and their applicability for precision medicine. It is well known that tumors exhibit substantial phenotypic differences between and within patients that can be identified by imaging [1, 2]. A significant advantage of medical imaging is its ability to noninvasively visualize a cancer’s appearance, such as macroscopic tumoral heterogeneity at baseline and follow-up, for both the primary tumor and metastatic disease.

In clinical practice, tumors are also often profiled by invasive biopsy and molecular assays; however, their spatial and temporal pathologic heterogeneity limits the ability of invasive biopsies to capture their biological diversity or disease evolution [3]. Furthermore, repeated invasive tumor sampling is burdensome to patients, expensive and limited by the practical number of tissue sampling that can be undertaken to monitor disease progression or treatment response. By contrast, the non-invasive imaging phenotype potentially contains a wealth of information that can inform on the expression of the genotype, the tumor microenvironment and the susceptibility of the cancer to treatments [4, 5].

Radiologists are generating their diagnoses by visually appraising the images, drawing on past experience and applying judgment. They perceive and recognize imaging patterns and infer a diagnosis consistent with the observed patterns [6]. Not surprisingly, there is a degree of subjectivity and variability in image interpretation. In an effort to reduce this variability, alternative approaches such as quantitative imaging have been proposed, wherein principle, it is possible to interrogate the images beyond what the naked eye can see and record measurements that are more objective. However, one of the most crucial obstacles for quantitative imaging at the moment is the fact that current scanners are designed to produce pretty pictures rather than as measurement devices, and there is limited ability to standardize the way of acquiring imaging data [7].

Tissue biopsy remains the primary source of information when it comes to tumor classification and staging. It is well known that biopsy is prone to sampling errors. Therefore, it may not be the best way to study tumor biology, given the fact that cancers are typically heterogeneous. On the other hand, imaging provides an opportunity to extract meaningful information of tumor characteristics in a non-invasive way. However, most derived images lack microscopic resolution, there is ionizing radiation exposure for some of the modalities, radiological assessment can be subjective, extracting quantitative parameters is time consuming and the results of these can vary significantly according to the imaging protocol with low reproducibility.

Hence, there is a clear need to improve the reproducibility and diagnostic accuracy of quantitative imaging features, and these have been the main driving forces for the development of radiomics, where we aim to associate quantitative voxel-wise imaging features with clinical outcomes and/or disease classification. Machine learning methods are increasingly applied to build, train, and validate models that can aid in the prediction of disease and treatment outcomes, as well as patient stratification, which is at the heart of precision medicine [8]. In the current review, we present an overview of the considerations and best practice for the development of radiomic signatures that can bring clinical value to the oncologic patient.

## Pipeline for the development of a Radiomic signature

**Fig. 1:**
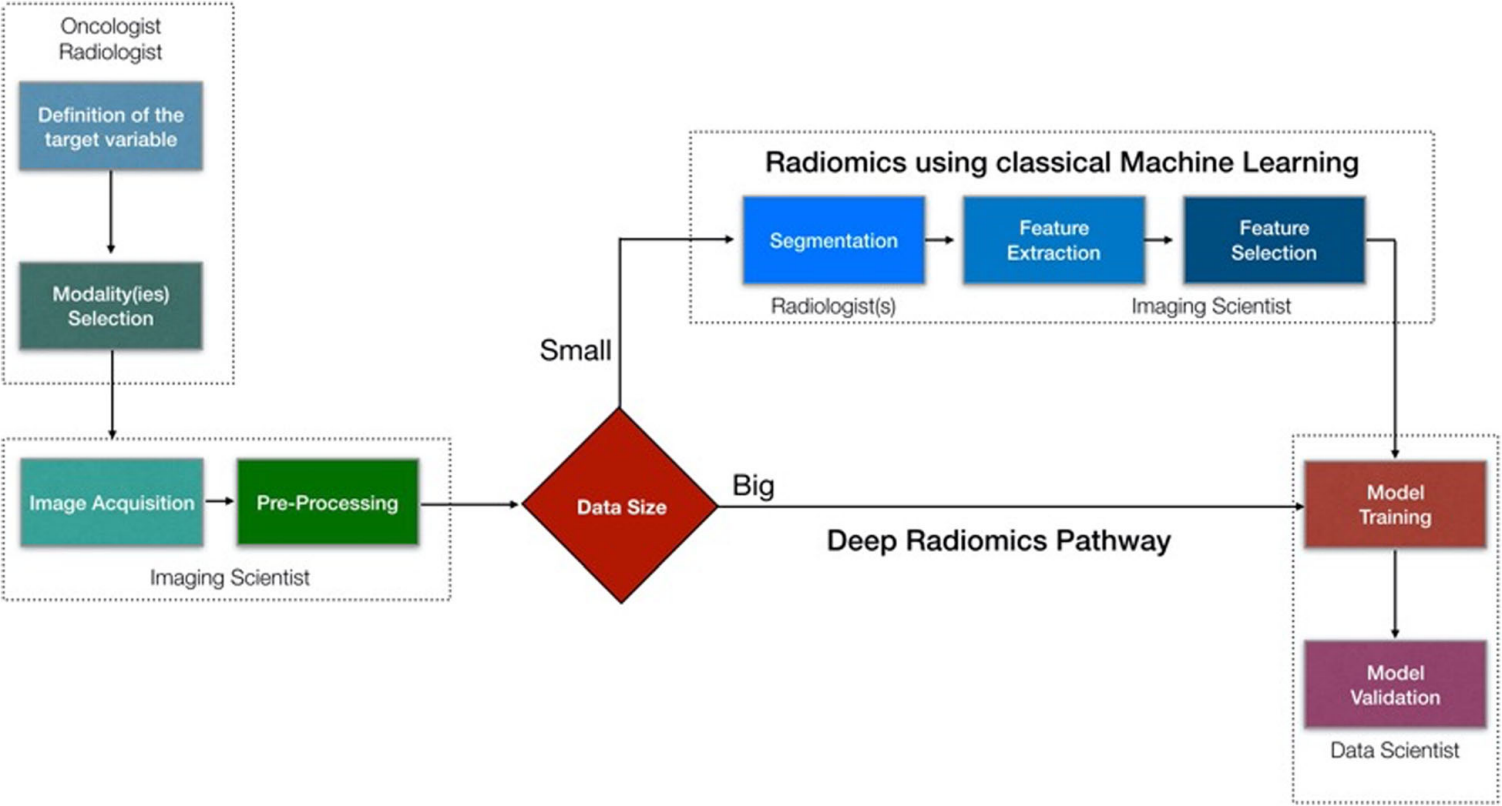
A multidisciplinary radiomics workflow. Initially a group of clinicians should define the clinical problem that the proposed model should deal with and make decisions on what kind of imaging modalities should be recruited. Imaging scientists needs to make sure that acquisition protocols are optimally designed producing high quality images, as well as for the pre-processing of the images. Then depending on the size of the available imaging studies we need to decide which pipeline to use. In case of big data (in the order of thousands) a deep radiomics approach can be suggested avoiding tedious and time-consuming processes like tumor segmentation by multiple radiologists. In addition, deep convolutional neural networks have been proven more efficient to model complex problems compared with traditional machine learning algorithms, as long as data availability requirement is satisfied. Finally, the data sets are allocated for training, validation and testing purposes

A Radiomics investigational pipeline comprises several phases including i) defining the clinical question and targeting the appropriate patient cohort, ii) identifying the relevant imaging modalities for radiomics analysis, iii) optimizing and standardizing the acquisition protocols, iv) applying pre-processing prior to image analysis, v) performing lesion segmentation on the images, vi) extracting handcrafted or deep imaging features, vii) reducing the dimensionality of the generated data by feature selection methods, and finally viii) training and validating the radiomics model (Fig. 1).

### Defining the clinical question and targeting the patient cohort

All radiomics projects should be informed by an appropriate clinical question that is underpinned by a scientific hypothesis. The clinical question should seek to address a current unmet need in cancer management, where the successful generation of a radiomics signature could result in better patient stratification, treatment selection and/or defining disease outcomes.

Although sample size matters, data quality is of equal importance, as well as data diversity. It is surprisingly difficult even today, where imaging data appears to be widely available, to be able to collect and curate, high quality, comprehensive imaging data. The minimum number of patients that need to be recruited to develop a radiomics signature is strongly dependent on the complexity of the problem we are trying to address [9]. For example, disease detection challenges where there is sufficient image contrast resolution to discriminate normal from abnormal tissue are considered far more straightforward and therefore needing fewer patients compared with more complex problems such as predicting patient treatment response or disease-free survival.

From a purely statistical viewpoint, for a binary classification problem, 10 to 15 patients are required for each feature that is participating within the radiomic signature [10]. In other words, a 5-feature signature requires between 50 and 75 patients for model training purposes. Ideally, 25–40% of the training sample should be the size of an external validation patient group for testing the model to estimate its real diagnostic performance. Radiomics studies have been published with as few as 20–30 patients, making the results of such models highly questionable due to the risks of model overfitting and the high instability of such models.

Another important consideration of any radiomics project is the identification of the patient cohort that is representative of the target patient population in terms of disease prevalence or incidence. Clearly, for diseases with low prevalence or incidence, this approach may not be pragmatic as very large study populations may be needed to develop a radiomics signature as useful classification tool or for predicting disease outcomes. In such instances, a-priori knowledge of the class sizes of the different disease sub-types that will be categorized by the radiomics analysis will help to determine whether the investigation will be useful at the outset. If not, it will be necessary to enrich the datasets with sufficient cases of each of disease sub-type for meaningful analysis. On a practical level, many centers may be limited by the number of cases that are available for any radiomics analysis and it is important to appraise the validity of using these constrained datasets at the outset to avoid wasting time performing the full analysis without the likelihood of any meaningful conclusions.

Nonetheless, some degree of heterogeneity when selecting the study population is inevitable to meet the high demands of radiomics with regards to the optimal number of patients that should be included. However, there is a delicate balance between the latter and the risk of inefficient learning of the models due to increased data variability.

### Identifying the relevant imaging modalities for radiomics analysis

A significant issue in quantitative imaging studies is the high variability of the acquisition protocols and the lack of standardization [11, 12]. The fundamental design of imaging modalities by the vendors is based on the assumption that a human will interpret images through visual perception, which undoubtedly leads to problems not permitting imaging modalities to act as quantitative devices that produce standardized quantitative results. The contributing reasons for the latter is the fact that measured parameters on images vary depending on the vendor platform, the type of hardware and software available on the scanner, the radiographer conducting the examination, as well as the radiologist performing the imaging analysis. Hence, comparing results across institutions can be challenging. Furthermore, there are now hybrid imaging systems that can produce a wealth of different imaging contrasts, so careful selection of the type of images that should be exploited is important for meaningful results. For example, it is well acknowledged that CT data is less variable compared with MRI data; and for this reason, CT images are most widely used for radiomics analysis.

### Optimising and standardising the acquisition protocols

It is important to develop and follow standardized acquisition protocols that can ensure accurate, repeatable and reproducible results. The level of standardization is not possible to be estimated a priori; therefore, a trial and error approach should be utilized to define such requirements. It is almost impossible to know apriori for each specific problem what is the level of image acquisition standardization needed. It is advised to generally start using a more liberal approach accepting a certain amount of heterogeneity, and only if we can’t identify useful features or otherwise called “signal” in our models we introduce certain restrictions in the form of standardized protocols (vendor, sequence parameter, MRI field strength, and others). One way to look at historical changes related to the image acquisition protocols, competency of scanners that in principle is improving due to upgrades and updates is the so-called temporal validation [13]. According to the latter the model training is based on rather heterogeneous cohort of patients obtained through the years but testing of the model is done exclusively with recently acquired exams. In this way the model becomes robust to changes related to the past by “seeing” diverse examples, although the disadvantage of such an approach is the higher number of patients needed. In case of multi-centric studies or in the event of multi-vendor single center studies there are two strategies. Either train with data from one site (or vendor) and test with data from the other sites (or vendors) or use mixed data to do both training and validation. Again, which of the two strategies is more effective needs to be proved by trying both and deciding on the basis of the highest performance and generalizability [14, 15].

### Applying pre-processing prior to image analysis

**Fig. 2:**
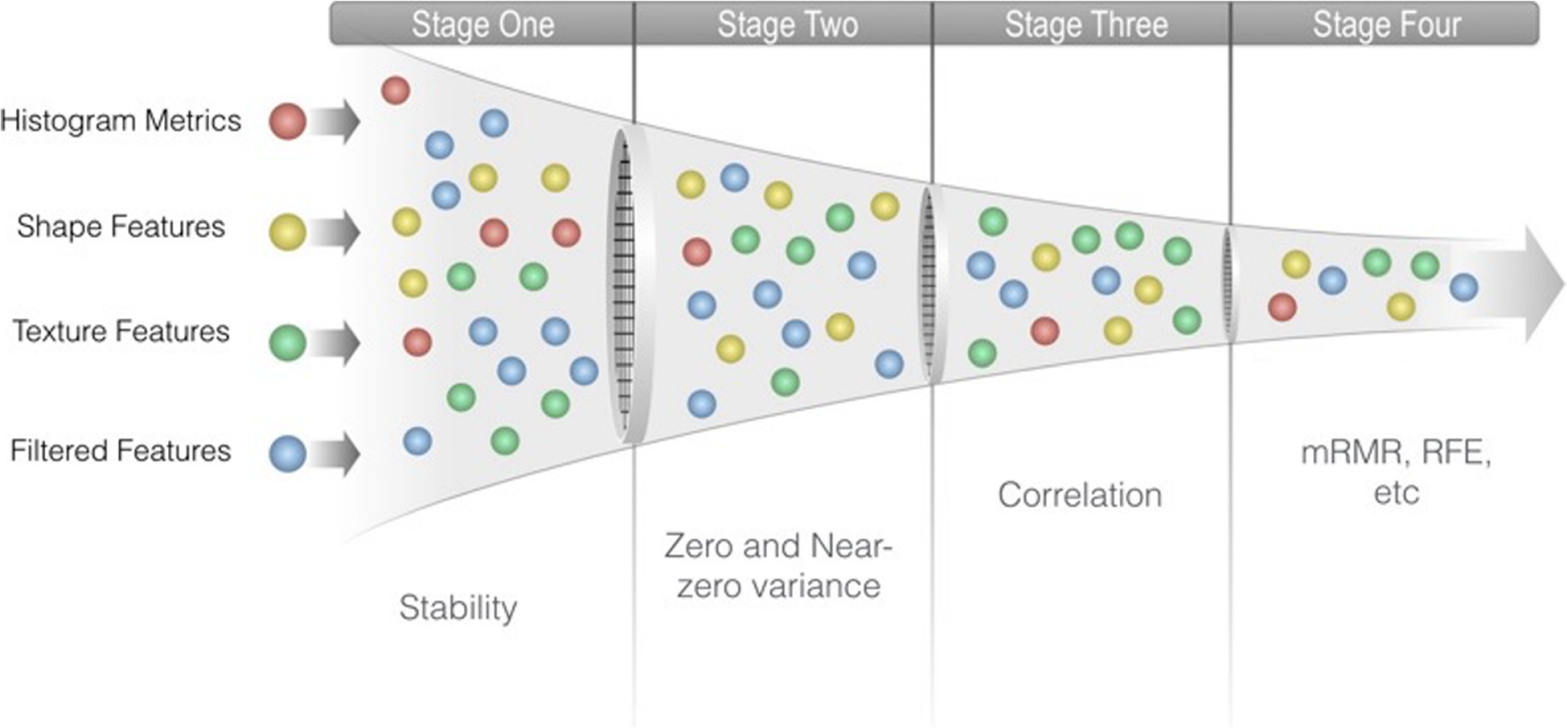
Feature selection is accomplished by applying several methods in a cascade manner. A typical workflow in the first phase permits only stable features to be forwarded, then a zero or near-zero variance method is removing useless features, then a correlation analysis is removing redundant features and finally a more sophisticated method like maximum relevance minimum redundancy (mRMR) or recursive feature elimination (RFE) is used to craft the final Radiomic signature

Preprocessing, including improvement of data quality by removing noise and artifacts, can improve the performance of the final models since the “garbage in – garbage out” concept applies in Radiomics [16]. Different kinds of filtering methods can be recruited to remove image noise but these needs to be used carefully since there is a risk of losing “signal”. Motion correction methods can cope with 4D data like in CT of the lung [17], diffusion-weighted imaging or dynamic contrast-enhanced MRI to remove patient motion from several image acquisitions. Filtering and motion correction techniques should be used only as a last resort, and effort should be undertaken in the image acquisition phase to eliminate the need of such invasive and in principle destructive methods. MRI images are known to suffer from spatial signal heterogeneities that cannot be addressed to biological tissue properties, rather than to technical factors like the patient body habitus, the geometric characteristics of external surface coils, the rf pulse profile imperfections and so on. Therefore, it is highly recommended that bias field correction algorithms [18] should be applied to remove such spatial signal heterogeneity (Fig. 2). Signal normalization is necessary to bring signal intensities to a common scale, without distorting differences in the ranges of values. Especially in the setting of heterogeneous data coming from different vendors or different acquisition protocols normalization is critical for the training process. It improves the numerical stability of the model and often reduces training time and increases model performance [19].

### Performing lesion segmentation on the images

After successful image acquisition and preprocessing, the next phase in the radiomics pipeline is to perform lesion segmentation in one or all slices containing the target lesion. The basic approach involves manual tracing of the lesion borders that might have high inter-reader variability, which can result in the derivation of unstable radiomic features. In order to build more robust models, stable features should be identified. One way of identifying feature stability is to perform at least two radiological segmentations on the same lesion, which are then analyzed to identify the stable features using simple correlation analysis [20].

**Fig. 3:**
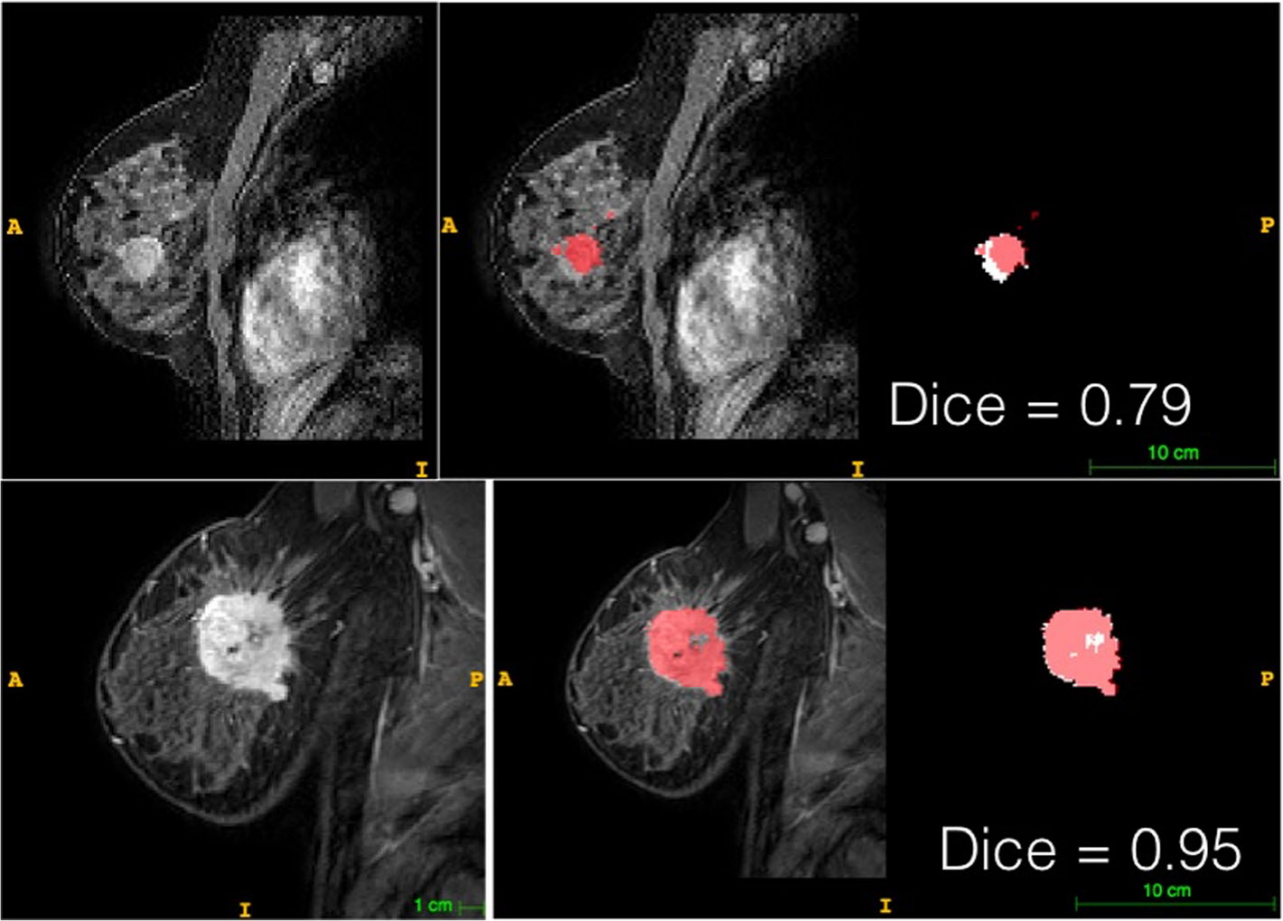
A convolutional neural network (VNet architecture) was trained on arterial phase images of a dynamic contrast enhanced MRI dataset to automatically segment enhancing breast lesions. Two examples are shown (the worst and the best) with an average DICE coefficient of 0.82 ± 0.15. The pink color denotes the pixels that where considered from the network as a lesion while the white pixels where corresponding to the radiologists’ segmentation used as the ground truth. The DICE coefficient is defined as 2 * the Area of Overlap between the pink and white areas divided by the total number of pixels in the segmentation mask. 130 patients in total were recruited for the training of the model

Currently, automatic disease segmentation is an active research field [21–26], which can potentially reduce inter-reader variability, as well as reducing the work burden on radiologists to under these tasks, thereby making the analysis large data sets more viable (Fig. 3). However, even with automatic disease segmentation, it is important for board-certified radiologists to verify and approve the final segmentation result.

A more recent approach is the delineation of the different physiologically distinct regions (e.g., blood flow, cell density, necrosis, and edema) within the tumor, also known as habitats [5, 27] which can be based on using functional imaging measurements, especially MRI. The radiomic features can then be extracted for each of these habitats. It is also interesting that recent studies have also found that radiomics features in peri-tumoral regions can also provide novel information that informs on treatment effects and disease outcomes.

### Extracting handcrafted or deep imaging features

The primary objective of radiomics is to provide comprehensive assessment of the imaging phenotype using automated data extraction algorithms. The latter can be served by calculating a large number of quantitative imaging features that capture a wide variety of phenotypic traits. Radiomic features can be classified into agnostic and semantic [2]. Semantic features are commonly used by radiologists to describe lesions like diameter, volume, morphology, while agnostic features are mathematically extracted quantitative descriptors, which are not part of the radiologists’ lexicon. These features are identified by algorithms that capture patterns in the imaging data, such as first-, second-, and higher-order statistical determinants, shape-based features, and fractal features. First-order statistics can be used to describe voxel values without concern for their spatial relationships. These can be used to quantify phenotypic traits, such as overall tumor intensity or density (mean and median of the voxels), or variations (range or entropy of the voxels). There is also shape- and location-specific features that capture 3-dimensional shape characteristics of the tumor. Second-order statistical features takes into account the spatial relationships of the image contrast between voxels. They are also referred to as texture features [28]. Texture is defined as “a regular repetition of an element or pattern on a surface with the characteristics of brightness, color, size, and shape.” Examples of texture features include the gray-level co-occurrence matrix, gray-level dependence matrix, gray-level run-length matrix, and gray-level size zone matrix [29]. These matrices describe textural differences based on grey tone spatial dependencies. Advanced methods, such as wavelet and Laplacian of Gaussian filters, can be applied to enhance intricate patterns in the data that are difficult to quantify by eye. When using artificial neural networks, the so called “deep” features can be extracted during the training phase, which are very powerful for mapping non-linear representations when there is adequate training data volume. However, deep features suffer from low interpretability, acting as black boxes and are therefore treated with variable sceptisism because they are difficult to conceptualise; compared with engineered or semantic features, which are often associated with biological underpinnings.

### Reducing the dimensionality of the generated data by feature selection methods

The high-dimensional nature of radiomics makes it sensitive to the so-called “curse of dimensionality” being responsible for model overfitting [30]. It is critical to minimize overfitting in order to build robust radiomic signatures that are generalizable and are robust to detect differences between new patients not used for training the model. Various strategies have been proposed to minimize overfitting. The most obvious is to train the models with more data since there is a reverse linear relationship between overfitting and the amount of data used for training. In case the latter is not feasible, dimensionality reduction is critical to be achieved through feature selection/reduction methods.

Feature selection methodologies can identify redundant, unstable, and irrelevant imaging features and remove them from further analysis. In this way, the few highly informative, robust features constituting the “signal” of the model will be employed in constructing a robust radiomic signature [31–34].

Feature stability can be assessed for consistency in the test-retest setting, the so-called temporal stability; or in terms of robustness of features to variations in tumor segmentation the so-called spatial stability [31]. Another type of stability check that needs to be considered in case of a split-validation scheme is to compare the distribution of values of each radiomic feature in the training and test datasets, keeping only those that show no significant difference between the two to avoid outliers or systematic errors between the training and test sets. It is worth keeping in mind that this type of feature selection is unsupervised since we do not need to reveal the ground truth variable during the process.

**Fig. 4:**
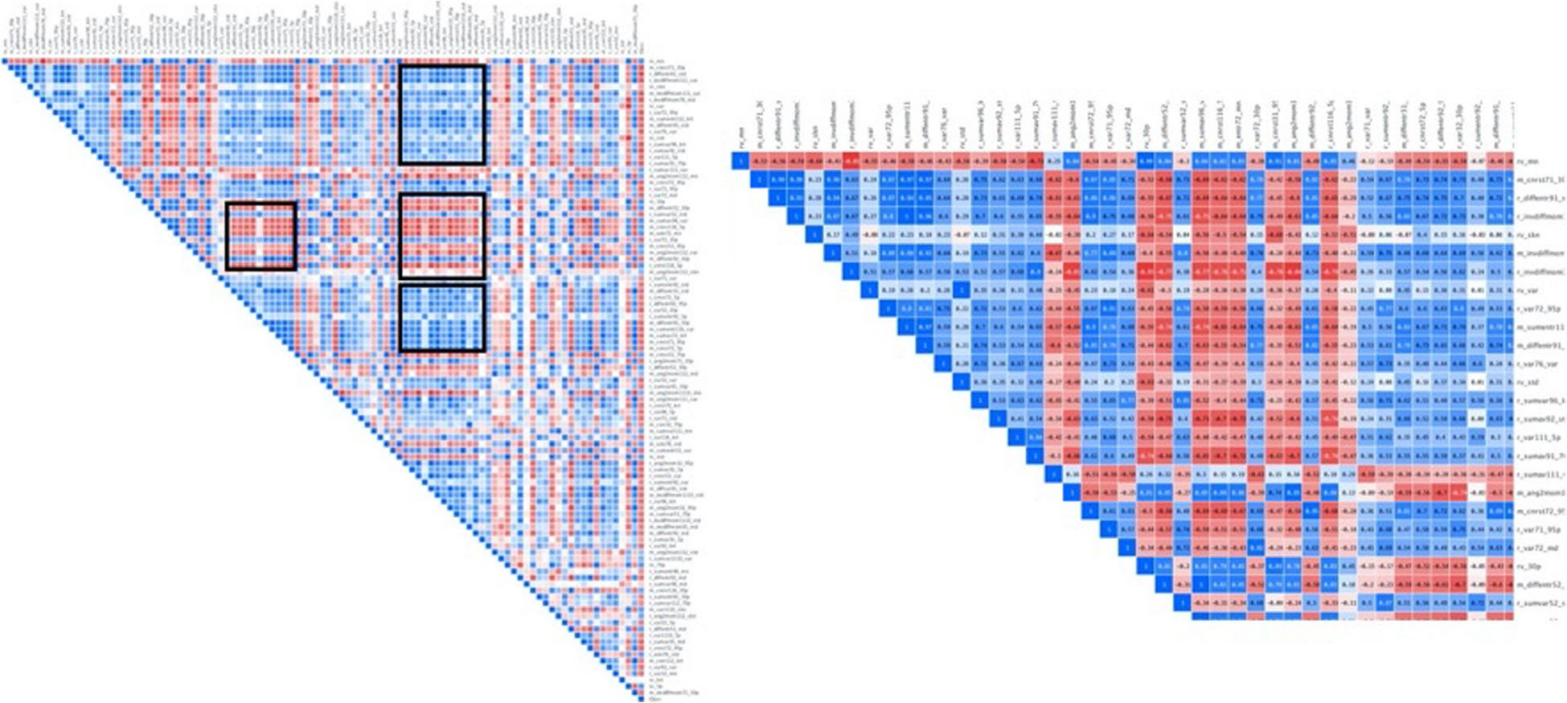
Correlation analysis heatmap showing blocks of highly correlated radiomic features (black frames on the left and positive with red color or negative correlation with blue color on the right). When identifying such groups of highly correlated features all but the one with the highest variance are removed from further analysis. In this case, the correlation coefficient was set to 95%

**Fig. 5:**
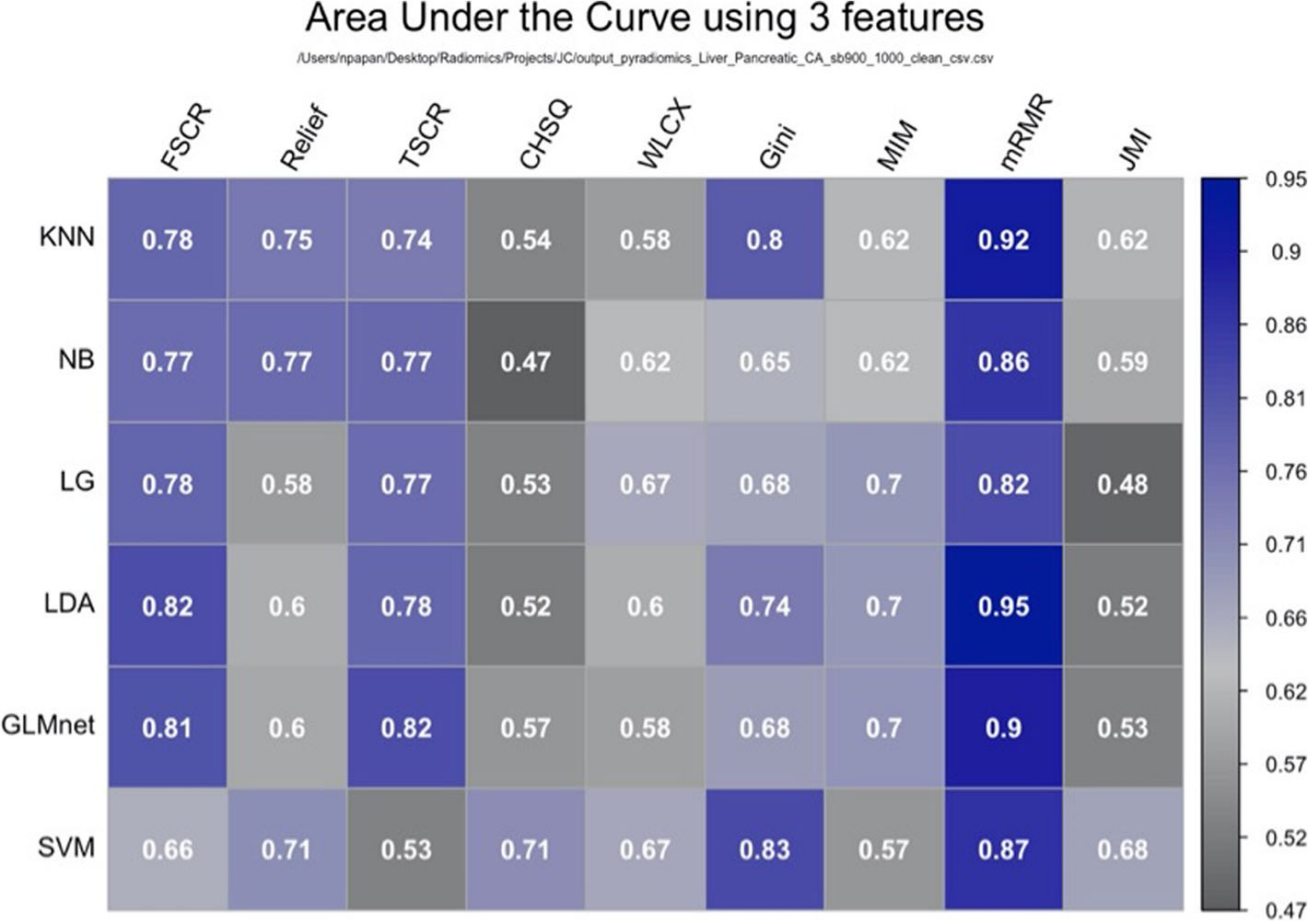
A heatmap aggregating the performance results of combinations of 6 machine learning models and 9 feature selection techniques. The dataset used for this analysis comprised features extracted from malignant pancreatic neoplasms on diffusion-weighted MRI acquired at high b value images (? of b = 900 s/mm^2^), which is used to distinguish patients with synchronous liver metastases from those without metastases. The best performing combination was an LDA model with mRMR feature selection method. SVM: Support Vector Machine, GLM: General Linear Model, LDA: Linear Discriminant Analysis, LG: Logistic Regression, NB: Naïve Bayes, KNN: K Nearest Neighbor, FSCR: Fisher Score, TSCR: T-Score, CHSQ: CHI-Square, WLCX: Wilcoxon, Gini: Gini index, MIM: Mutual Information Maximization, mRMR: minimum Redundancy Maximum Relevance, JMI: Joint mutual information

Following the identification of stable features, we need to remove redundant features using a correlation-based feature elimination method [32]. After constructing a correlation heatmap, we can identify blocks of features (Fig. 4) that presents with a correlation coefficient being higher than a predefined threshold (i.e., 95%) and remove them. Now that a significant number of features have been removed, we proceed with more sophisticated methods in order to further reduce the dimensionality and construct our radiomic signature that will comprise a few features. For the latter task, different methods can be used. These methods can be divided into three categories, namely filter, wrapper, and embedded methods. Filter methods perform feature ranking, the selection is based on statistical measures and are characterized by their computational efficiency, generalization, and robustness to overfitting. Filter methods can either score features independently (univariate methods), by ignoring the relationship between features or take into account the dependency between features (multivariate methods). Univariate filter methods are usually used as a preprocessing step since redundancy is not analyzed. In wrapper methods, searches to identify subsets of relevant and non-redundant features are performed, and each subset is evaluated based on the performance of the model generated with the candidate subset. These methods are susceptible to overfitting and are computationally expensive. The embedded methods perform feature selection and classification simultaneously, taking advantage of their feature selection methods and learning, which create more accurate models. By comparison with wrapper methods, embedded methods are computationally efficient [33]. Usually, we construct heatmaps showing the performance of using the different machine learning models with various feature selection methods (Fig. 5).

### Training and validating the radiomics model

Following feature selection, a set of non-redundant, stable, and relevant features can be used to develop a model that will try to answer the selected clinical question, which is also called ground truth or target variable. Depending on whether the result of the clinical question is a continuous or a discrete variable, different methods should be used. When working with continuous variables, regression methods, such as Linear Regression, Cox (Proportional Hazards), Regression Trees, or others can be used. As for discrete variables, we can use classification methods such as Logistic Regression, Naïve Bays, Support Vector Machines, Decision Trees, Random Forests, K-nearest neighbors and others [34]. To estimate the performance of the trained model, we should ideally have two distinct patient cohorts. The larger should be used for training and fine-tuning the model, while the smaller, ideally from a different institution, should be used to validate the model. The latter provides external validation that will result in more realistic estimates of the model’s performance. This ensures that we able to develop radiomics signatures that can be applied across clinical settings. Unfortunately, very few published models are developed using external validation counting, on average, 6%, according to a recent study that evaluated 516 published models [35]. The vast majority of the published models are based on single-institution retrospective patient cohorts using the so-called internal validation. Using the latter approach, the study cohort is divided into two different subsets, the training subset is used to develop the model, while the testing subset is used only for the validation and evaluation of the model derived from the training subset. In the case of a very small dataset (between 50 and 100 patients), the internal validation approach has a significant risk of bias since a single test set comprising a few dozen data points (i.e., 20–30 patients) can easily provide over optimistic or pessimistic estimates of model performance.

**Fig. 6:**
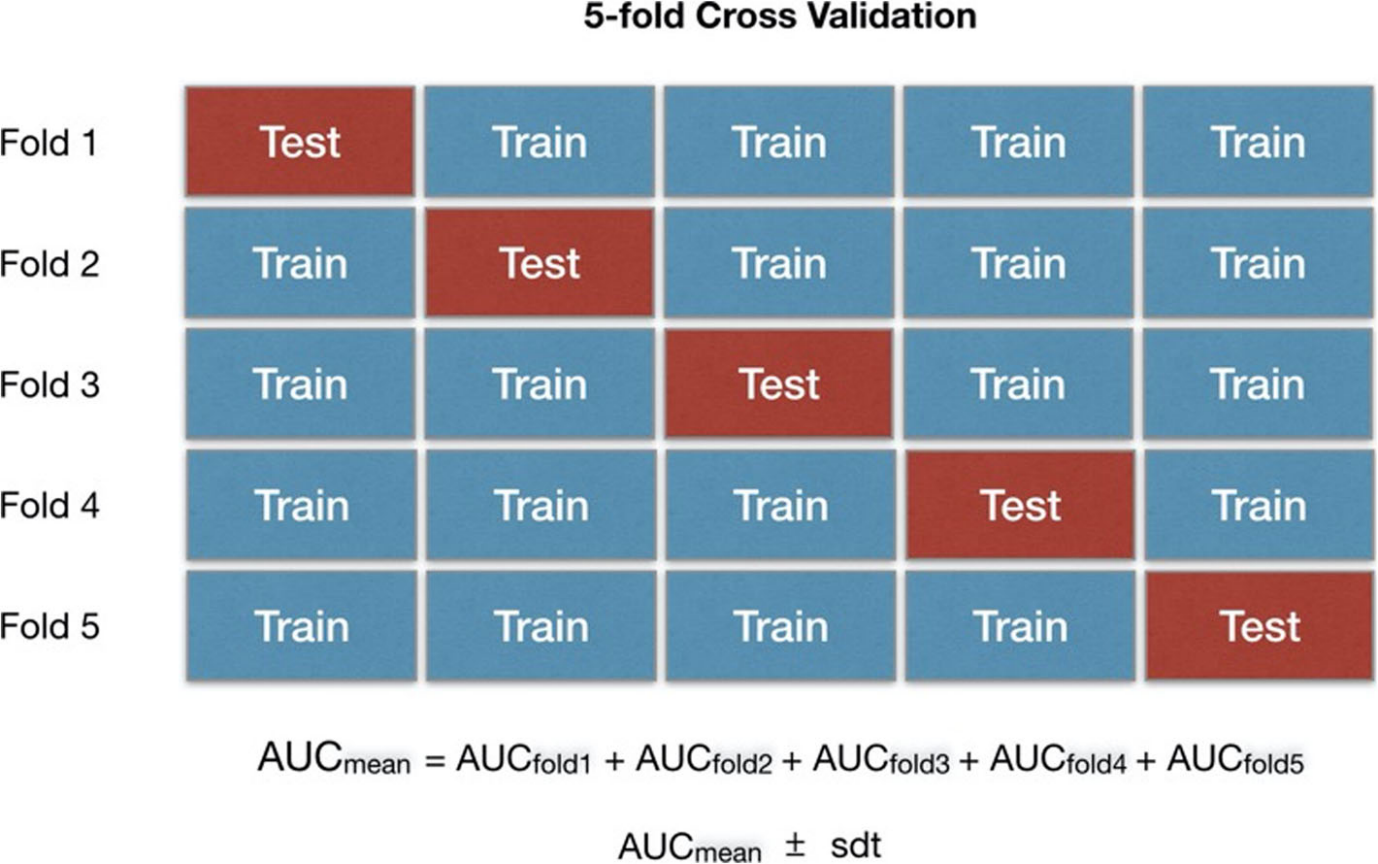
Resampling scheme based on the total dataset and iterative multi-splitting scheme based on a 5-fold cross validation. Apart the performance metric (mean AUC in that case) it is of equal importance to report the standard deviation across the folds to get an indication of robustness of the model. Low standard deviations are reflecting stable and robust models that are not influenced by the specific test set

One way to deal with this problem is to utilize a cross-validation approach that comprises the separation of the small cohort into multiple training and testing sets [33]. In k-fold cross-validation, the original sample is randomly partitioned into k equal sized subsamples (Fig. 6). Of the k subsamples, a single subsample is retained as the validation data for testing the model, and the remaining k − 1 subsamples are used as training data. The cross-validation process is then repeated k times (the folds), with each of the k subsamples used exactly once as the validation data. The k results from the folds can then be averaged to produce a single estimation. Apart from the average performance of a model, the standard deviation computed across the folds should be reported since that is a measure of the model’s reproducibility and robustness. It is often the case when comparing multiple models, to choose the one with slightly inferior performance but significantly lower standard deviation since this model will be potentially more robust and reproducible.

There are two main types of learning schemes in radiomics; the supervised learning where the model development is based both on input (radiomic features) and output data (ground truth variable), and the unsupervised learning approach where the model is trying to reveal potential associations or correlations using only the input data. A typical example of the latter is the hierarchical cluster analysis that has been applied to find associations between imaging features and gene expressions in the so-called radiogenomic models, where several sets of imaging features may be observed in association with specific gene expressions.

In general, radiomic features can be investigated alongside many other -omics types of data, including proteomics, metabolomics, and others. Integration of multiple, diverse sources of data using different kind of fusion strategies either at a feature or at a model decision level is a current trend in predictive modeling. In particular, it is very common to add radiomic features to clinical variables that are predictors of the disease outcome in the form of nomograms [36–38], which can then be applied and tested within clinical cohorts.

## Conclusions

The impetus for developing a radiomics signature often arise from unmet clinical needs in disease detection, characterization, staging, as well as prediction of treatment response and disease survival. The approach to radiomics should follow best practices not only generic to data science but also take into consideration domain related conditions, particularly when dealing with smaller datasets that are frequently encountered in cancer imaging. Optimal radiomics analysis in cancer imaging requires a multidisciplinary approach, involving expert knowledge of Oncologists, Radiologists, Imaging Scientists and Data Scientists. There is a cogent need for radiologists to drive these projects through domain knowledge which has key influence on many parts of the workflow and the eventual outcome.

## Data Availability

Not applicable for a review paper.
